# Impact of the Combination of Epigallocatechin Gallate and Ellagic Acid Supplemented with Ketone Bodies on Energetic Restoration of Mitochondrial Dysfunction and Metabolic Inefficiencies in Patients with Multiple Sclerosis: A Review

**DOI:** 10.3390/ijms27052168

**Published:** 2026-02-25

**Authors:** Jose Enrique de la Rubia Ortí, Alba Roig-Soriano, Sandra Carrera-Juliá, Alejandra Castelló-Guillen, Marisa Machado, Rocío García-Villalba, Jorge Alarcón-Jiménez, Nieves de Bernardo, María Benlloch

**Affiliations:** 1 Department of Nutrition and Dietetics, Catholic University of Valencia San Vicente Mártir, 46001 Valencia, Spain; joseenrique.delarubi@ucv.es (J.E.d.l.R.O.); alba.roig23@mail.ucv.es (A.R.-S.); alejandra.castello@mail.ucv.es (A.C.-G.); 2 UCIBIO—Applied Molecular Biosciences Unit, Translational Toxicology Research Laboratory, University Institute of Health Sciences (1H-TOXRUN, IUCS-CESPU), 4585-116 Gandra, Portugal; sonia.marisa@ipsn.cespu.pt; 3 Associate Laboratory i4HB—Institute for Health and Bioeconomy, University Institute of Health Sciences—CESPU, 4585-116 Gandra, Portugal; 4 H^2^M—Health and Human Movement Unit, Instituto Politécnico de Saúde do Norte, Cooperativa de Ensino Superior Politécnico e Universitário (CESPU), CRL, 4760-409 Vila Nova de Famalicão, Portugal; 5 Quality, Safety and Bioactivity of Plant-Derived Foods, Centro de Edafología y Biología Aplicada del Segura-Consejo Superior de Investigaciones Científicas (CEBAS-CSIC), 30100 Murcia, Spain; rgvillalba@cebas.csic.es; 6 Department of Physiotherapy, Catholic University San Vicente Mártir, 46001 Valencia, Spain; jorge.alarcon@ucv.es (J.A.-J.); nieves.debernardo@ucv.es (N.d.B.); 7 Department of Basic Biomedical Sciences, Catholic University of Valencia San Vicente Mártir, 46001 Valencia, Spain

**Keywords:** multiple sclerosis, epigallocatechin gallate, ellagic acid, ketone bodies

## Abstract

Multiple sclerosis (MS) is characterized by progressive mitochondrial dysfunction affecting complexes I, III, and IV of the electron transport chain, contributing to axonal energy failure and neurodegeneration. This review examines the potential of combining β-hydroxybutyrate (βHB), epigallocatechin-3-gallate (EGCG), and ellagic acid (EA) as a multi-target therapeutic strategy to restore mitochondrial function in patients with MS. Experimental and clinical studies demonstrate that each compound exerts complementary mechanisms. Ketone bodies provide an alternative energy substrate and restore complex I activity via sirtuin-dependent pathways. EGCG acts predominantly at the peripheral level by reducing systemic inflammation and oxidative stress. EA-derived urolithins effectively cross the blood–brain barrier to directly enhance mitochondrial biogenesis and respiratory chain function in the central nervous system. Clinical trials have reported improvements in fatigue, cognition, mood, and muscle function following supplementation with these compounds. The convergence of their actions on energy restoration, reactive oxygen species reduction, and epigenetic modulation of protective pathways suggests their synergistic potential. Optimized delivery strategies, including exogenous ketone salts, liposomal EGCG, and microencapsulated EA, may overcome bioavailability limitations and interindividual variability in the gut microbiota metabolism.

## 1. Introduction

Multiple sclerosis (MS) is a neurodegenerative disease characterized by damage to the myelin sheath covering neurons and is the main cause of disability in young adults [1]. Currently, there is no medical cure for MS, which is the main variety of relapsing–remitting MS, and fundamentally presents with fatigue, functional disability, muscle weakness, optic neuritis, and cognitive and emotional problems that have a significant impact on quality of life and disease prognosis [2].

Three pathogenic mechanisms have been proposed to explain this neuronal alteration: (a) excessive accumulation of intra-axonal Ca^2+^ [3]; (b) demyelination of axons that evolves into a degenerative process due to lack of trophic support provided by myelin or myelin-forming cells [4]; (c) inflammatory processes mediated by alterations in the immune system [5,6,7].

These alterations modify mitochondrial activity in the axons, leading to a decrease in ATP concentration, which has a drastic effect on axonal damage. Various evidence indicates that complexes I, III, and IV of the electron transport chain (ETC) are the main points of mitochondrial dysfunction in MS, both in demyelinated lesions and in apparently normal gray matter. The mitochondrial dysfunction leads to a lack of energy in nerve fibers (axonal energy deficits), encouraging a rise in cellular damage (increased oxidative stress) and gradual nerve decline (progressive neuroaxonal degeneration). These outcomes are typical of the stages of chronic disease [8,9].

Complex I (NADH dehydrogenase) is usually the most affected complex in the disease. Histochemical and biochemical studies in cortical and spinal cord tissues from patients with MS show a significant reduction in its activity in demyelinated axons, along with loss of subunits encoded by mitochondrial DNA (NDUFA9, ND6), mitochondrial fragmentation, and decreased membrane potential [10]. This dysfunction limits electron entry into the respiratory system and favors the escape of reactive oxygen species (ROS).

Complex III (ubiquinol–cytochrome c oxidoreductase) also shows functional and structural alterations, as well as an increase in the oxidation of its lipid components [11]. These alterations reduce electron transfer toward cytochrome c and amplify superoxide radical production, contributing to secondary mitochondrial injuries.

Finally, complex IV (cytochrome c oxidase, COX) is compromised in both the oligodendrocytes and neurons. A reduction in the expression and activity of COX-I and COX-IV subunits has been described in chronic cortical lesions and adjacent normal white matter. This deficit could be due to oxidative and nitrosative damage induced by activated microglia, which inactivate the heme-copper center of the enzyme [12]. Loss of complex IV function compromises oxidative phosphorylation and precipitates axonal energy failure, which is one of the main mechanisms of neurodegenerative progression in patients with MS.

Together, the coordinated alteration of complexes I, III, and IV deteriorates ETC efficiency, promoting mitochondrial superoxide formation, inner membrane depolarization, and release of pro-apoptotic factors.

For the functional restoration of these complexes, metabolic strategies, such as increasing ketone bodies or administering polyphenolic antioxidants, could be a potential alternative.

The present review aims to analyze the mechanistic basis and clinical evidence supporting the combined use of ketone bodies and different polyphenols as a complementary strategy for mitochondrial restoration in patients with MS.

## 2. Ketone Bodies: Energetic Alternative in Multiple Sclerosis

Ketone bodies, mainly β-hydroxybutyrate (βHB), which is the most abundant and biologically relevant [13,14], have shown beneficial effects on mitochondrial function and neuronal bioenergetics. Specifically, it has been observed that in cellular models with complex I deficiency, supplementation with ketone bodies partially restored the activity and assembly of said complex, increased mitochondrial biogenesis, and normalized the NADH/NAD^+^ ratio [15,16], which was also observed in an animal model [17] of Amyotrophic Lateral Sclerosis. This effect is associated with the activation of sirtuin (SIRT)-dependent pathways [18], such as SIRT3, and mitochondrial biogenesis coactivators, which promote the repair of damaged complexes [19]. In this sense, primary motor neurons increase SIRT expression after treatment with medium-chain triglycerides (MCTs) as a source of ketone bodies; therefore, these metabolites could regulate mitochondrial activity and cell survival through SIRT-mediated responses [18]. Interestingly, SIRT3 also regulates ketone body production, which is confirmed by the elevation of SIRT expression in primary motor neuron cultures after MCT treatment. Furthermore, ketone bodies can act as alternative metabolic substrates, by bypassing complex I and feeding electrons directly into complex II via succinate oxidation [20,21].

However, it is important to consider that neuroinflammation induced by metabolic hypoxia refers to an inflammatory state within the central nervous system (CNS) driven by insufficient cellular energy availability despite preserved oxygen supply. This is particularly relevant in MS, as demyelination markedly increases axonal energy demand owing to the loss of saltatory conduction, leading to mitochondrial dysfunction, ATP depletion, and increased reactive oxygen species (ROS) production. This condition, often described as metabolic hypoxia, contributes to axonal injuries and neurodegeneration [22]. In this context, ketone bodies, particularly βHB, can attenuate neuroinflammation induced by metabolic hypoxia by serving as efficient alternative energy substrates and enhancing mitochondrial bioenergetics [14].

## 3. Possible Dual Action of Different Polyphenols: Epigallocatechin Gallate and Ellagic Acid in Multiple Sclerosis—Microbial Activity and Most Active Metabolites

Polyphenols, such as epigallocatechin-3-gallate (EGCG), the principal catechin in green tea, and ellagic acid (EA), primarily found in pomegranates, berries (raspberries, strawberries, blackberries), and walnuts, have emerged as modulators of oxidative stress and neuroprotection. This effect could be relevant from an energetic perspective at the mitochondrial level in MS improvement.

In this sense, treatment with EGCG has been studied in an animal model of MS (experimental autoimmune encephalomyelitis (EAE)) and in neuronal cells in vitro. This treatment preserves complex I activity by reducing NADH dehydrogenase oxidation [23,24]. It also modulates complex III, decreasing superoxide release and maintaining the mitochondrial membrane gradient [25]. In addition, it indirectly improves complex IV efficiency by reducing oxidative stress and inflammation [26]. However, owing to its low permeability through the blood–brain barrier (BBB), which is 2–3%, direct action on neuronal mitochondria is limited. Its main effect is observed on peripheral immune cells and the cerebral endothelium, where it reduces the production of proinflammatory cytokines, which, in turn, decreases secondary mitochondrial injury [27]. This fundamentally peripheral effect could justify the important results achieved in our laboratory, especially at the motor level, where it was observed that after 4 months of treatment with EGCG (800 mg daily), patients diagnosed with MS had significantly increased muscle percentage accompanied by a decrease in IL6 [28] and significantly decreased cardiac risk [29], functional disability [30], and fat percentage, which were associated with an increase in albumin and paraoxonase 1 (PON1) [31]. These improvements in muscular, motor, oxidative stress, and inflammatory levels could explain the lower perception of anxiety [32] and depression [33] that was also observed after the intervention.

To deepen the analysis of EGCG efficacy, it is essential to identify the most effective metabolites. After ingestion, EGCG undergoes rapid intestinal metabolism. Only a small fraction circulates in free form, while most components are transformed into phase II metabolites (glucuronides, sulfates, and methylated derivatives) and products resulting from microbial degradation of the flavanic ring in the colon. Among these latter, phenolic γ-valerolactones stand out, particularly 5-(3,5-dihydroxyphenyl)-γ-valerolactone and 5-(3,4-dihydroxyphenyl)-γ-valerolactone, currently considered the metabolites with greatest physiological relevance. These valerolactones exhibit anti-inflammatory activity, modulate glucose metabolism, including promoting GLUT4 translocation in skeletal muscle, and possess neuroprotective potential, possibly due to their greater stability than intact EGCG [34,35,36]. In contrast, EGCG conjugates (especially glucuronides and sulfates) reach higher plasma concentrations than the native molecule and can contribute to their antioxidant and anti-inflammatory effects, although their biological potency seems lower than that observed for γ-valerolactones [37].

γ-valerolactones preserve the mitochondrial membrane potential and reduce the production of reactive oxygen species (ROS) in mitochondrial dysfunction models [38].

Regarding their capacity to cross the BBB, EGCG-derived metabolites show capacity to cross the BBB and exert neuroprotective effects [28] by inhibiting microglial activation and reducing the production of proinflammatory cytokines [39,40]. However, this activity is limited, showing low cerebral distribution, which could be due to their bipolar functional group having difficulties penetrating the BBB; bound to proteins, it is a large complex that cannot easily traverse cells or even capillary membranes and, therefore, has restricted distribution in the brain [41].

Overall, evidence suggests that the clinical benefits of EGCG consumption are mainly due to its microbial transformation and the bioactivity of valerolactones, rather than the original catechin, and its limited capacity to cross the BBB, exerting its effect fundamentally at the peripheral level.

On the other hand, EA, especially its urolithin metabolites (A and B), effectively cross the BBB and accumulate in neural tissue [42,43,44,45,46,47]. Specifically, it is known ellagitannins and EA present in foods such as pomegranates, walnuts, and red fruits have beneficial properties at the neurocognitive level [46]. It should be noted that these polyphenols have low bioavailability and are widely metabolized by intestinal microbiota, giving rise to urolithins, the most common of which are urolithin A, urolithin B, and isourolithin A (isoA). It is precisely these metabolites to which the beneficial central effects of ellagitannins and EA are attributed. Once absorbed, they undergo phase II metabolism, appearing in systemic circulation and in different tissues as glucuronide and sulfate derivatives [47], and are able to easily cross the BBB. A recent in vitro study demonstrated that urolithins A and B and isoA, in both original and conjugated forms, can cross the BBB [44]. In a study in mice administered urolithin A intraperitoneally, urolithin A and its sulfated derivative were detected in the brain tissue [45]. This ease of crossing the BBB explains the activity of this polyphenol and its metabolites. Specifically, urolithin A: (1) increases the expression and activity of complexes I and III, restoring oxidative phosphorylation and reducing ROS production [48]; (2) modulates complex IV, increasing cytochrome c oxidase activity and improving mitochondrial oxygen consumption [49]; (3) activates mitochondrial biogenesis pathways mediated by peroxisome proliferator-activated receptor gamma coactivator 1-alpha (PGC-1α) and Nuclear Respiratory Factor 1 (NRF1), promoting the functional recovery of damaged mitochondria [48,50]. These effects promote axonal protection and better neuronal survival at the cerebral level, particularly in regions affected by active demyelization. They may enhance neuronal and axonal resilience within the CNS, potentially contributing to neuroprotection in patients with MS, thus justifying results obtained to date in people diagnosed with MS who received 180 mg AE daily, where there was an increase in IL-4, brain-derived neurotrophic factor, and serotonin and a decrease in interferon-γ (IFN-ƴ), nitric oxide (NO), cortisol, and gene expression of indoleamine 2, 3-dioxygenase [51,52]. These results could, in turn, be associated with the improvement of anxiety, depression, and disability, which are also observed in patients with the disease after receiving EA [53].

Therefore, the intake of a combination of EGCG and EA polyphenols seems to offer a complementary approach to treating mitochondrial dysfunction in MS. EGCG acts fundamentally at the peripheral level by reducing immune activation and systemic inflammation, decreasing oxidative stress that indirectly affects neuronal mitochondria, and preserving the endothelial integrity of the BBB, which could potentiate the entry of active EA metabolites [27,54], and it exerts these effects through its antioxidant and anti-inflammatory actions on cerebral endothelial cells, including the reduction in oxidative stress, modulation of inflammatory signaling pathways, and preservation of tight junction proteins. In the context of MS, these mechanisms may limit pathological disruption of the BBB and immune cell infiltration without increasing nonspecific permeability. In this way, it maintains a regulated and functional endothelial barrier, facilitating the controlled entry and effective activity of small bioactive molecules such as EA and its metabolites within the CNS. In fact, urolithins have been shown to exert a significant impact by increasing the protein abundance of mitochondrial respiratory chain complexes, thereby enhancing mitochondrial biogenesis and the efficiency of oxidative phosphorylation [49]. This could protect neurons and oligodendrocytes.

## 4. Metabolic Synergy of Ketone Bodies, EGCG and AE on Mitochondrial Respiratory Complexes

When assessing the possible synergy of the combination of ketone bodies, EGCG, and AE, it is interesting to note how these three components act on different nodes of the respiratory chain. Ketone bodies, especially βHB, favor the efficient oxidation of NADH in complex I, reducing excess electrons that feed ROS generation and increasing the electrochemical gradient [55]. Furthermore, βHB and acetoacetate can directly feed complex II through the succinate cycle, thereby optimizing energy flow and ATP production [16]. EGCG, for its part, fundamentally stabilizes complex IV, protecting it from oxidative damage and preserving mitochondrial membrane potential [26]. Additionally, EGCG has an indirect effect on complex I by reducing excess NADH and ROS, which can complement the actions of ketone bodies in mitigating oxidative stress. EA and its urolithin metabolites, which can effectively cross the BBB, restore the activity of complexes I, III, and IV and promote mitochondrial biogenesis mediated by PGC-1α and NRF1 [48,49,50].

This convergence of mechanisms allows for the simultaneous targeting of the three main sources of mitochondrial dysfunction in MS: energy deficits, ROS accumulation, and impaired mitochondrial biogenesis. These complementary actions reflect the pharmacodynamic synergy between the peripheral and central compartments. EGCG primarily exerts its effects at the peripheral level by reducing T- and B-cell immune activation, inhibiting NF-κB signaling, and improving BBB integrity [56,57]. In contrast, AE and its urolithins act within the CNS, where they modulate microglial activation, stimulate selective mitophagy, and restore neuronal metabolism [58,59]. Furthermore, ketone bodies freely cross the BBB and provide an alternative and clean energy source, decreasing glucose dependence and reducing neuroinflammation induced by metabolic hypoxia [60,61].

Finally, regarding their direct effects on respiratory complexes, the three components converge to activate epigenetic pathways of mitochondrial resilience. Both EGCG and AE induce the activation of AMPK and SIRT1 [38,50,62], whereas βHB acts as an endogenous inhibitor of histone deacetylases (HDACs) [63,64]. SIRT1 and AMPK function as coupled metabolic sensors, acting on overlapping intracellular pathways to regulate mitochondrial biogenesis, oxidative stress, inflammatory control and cellular energy homeostasis [65].

**Figure 1:**
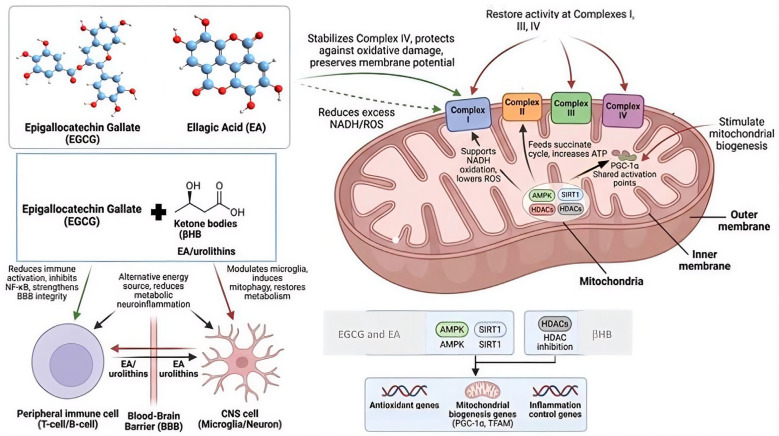
Possible metabolic synergy of βHB, EGCG, and EA on mitochondrial respiratory complexes in multiple sclerosis. EGCG acts predominantly at the peripheral level by reducing immune activation, inhibiting NF-κB signaling, and preserving BBB integrity. Ketone bodies freely cross the BBB to provide an alternative energy substrate, supporting NADH oxidation at complex I and feeding the succinate cycle at complex II. EA-derived urolithins cross the BBB and restore the activity of electron transport chain complexes while promoting mitochondrial biogenesis via PGC-1α and NRF1. These three compounds converge on epigenetic pathways through AMPK/SIRT1 activation and HDAC inhibition, enhancing the expression of antioxidant genes, mitochondrial biogenesis regulators (PGC-1α and TFAM), and anti-inflammatory mediators. Arrows indicate activation or promotion; dashed arrows indicate inhibition or reduction. AMPK (AMP-activated protein kinase), BBB (blood–brain barrier), βHB/AcAc (ketone bodies), EA (ellagic acid), EGCG (epigallocatechin gallate), HDACs (histone deacetylases), NF-κB (nuclear factor kappa B), PGC-1α (peroxisome proliferator-activated receptor gamma coactivator 1-alpha), ROS (reactive oxygen species), SIRT1 (sirtuin 1), TFAM (mitochondrial transcription factor A).

All these mechanisms are illustrated in Figure 1.

## 5. Possible Clinical Impact of Ketone Body Metabolites, EGCG, and EA in Patients with Multiple Sclerosis (MS)

To understand and justify the suitability of administering the three molecules, it is important to highlight previously published evidence demonstrating the role of these molecules and their microbial metabolites in improving various clinical aspects characteristic of pathology. Ketone bodies have been shown to modulate key inflammatory pathways and improve fatigue, cognition, and quality of life in individuals with MS [66]. Complementarily, caloric restriction, which promotes ketosis, has been found to exert early protective effects on vascular, cognitive, and mental health during aging [67].

Beyond these neurological and functional benefits, evidence suggests that ketosis plays a relevant role in preserving muscle function, as the ketogenic diet exerts a protective effect against muscle weakness by enhancing energy metabolism and stimulating muscle regeneration signaling [68]. Increased availability of ketone bodies, either through ketogenesis or parenteral infusion, protects against muscle weakness induced by chronic diseases [69]. In this context, supplementation with the ketone body βHB improves muscle strength in critically ill mice [70].

Moreover, findings indicate that this type of diet, when combined with medium-chain triglycerides, improves muscular dystrophy by inhibiting myonecrosis and promoting muscle stem cell proliferation in experimental animals (established rat models of Duchenne muscular dystrophy) [71]. Finally, it has also been observed that in critically ill patients admitted to an Intensive Care Unit, βHB levels are inversely correlated with fat area and associated with a lower risk of muscle wasting, confirming the protective activity of these metabolites at the muscular level [72].

In turn, the phenolic γ-valerolactones derived from EGCG have been shown to improve cognitive function and memory by promoting neuronal plasticity, suggesting a synergic effect of green tea in modulating the nervous system. The reviewed studies provide evidence that green tea influences psychopathological symptoms (e.g., reducing anxiety [33]), cognition (e.g., improving memory and attention), and brain function (e.g., activating working memory) [73].

Moreover, it has been observed that metabolites produced from EGCG, primarily through interactions with the gut microbiota, may alleviate depression [74]. It has also been demonstrated that certain natural supplements (including epigallocatechin-3-gallate) are effective in treating spasticity, fatigue, memory impairment, functional performance, and tremors [75].

Additionally, antioxidant interventions aimed at restoring muscular redox homeostasis with EGCG may improve muscle function and reduce weakness in patients with MS [76]. Along these lines, EGCG has also shown efficacy in attenuating neuronal and retinal damage [77], thereby improving visual processing in healthy animal models, which reinforces its potential application in the context of neuroinflammation and neurodegeneration, such as MS [78]. Furthermore, in murine models of experimental autoimmune uveitis, which shares immunopathogenic mechanisms with MS, green tea extract and its component EGCG have been shown to preserve visual function [79].

Urolithins A and B and isoA derived from EA metabolism have shown relevant effects in MS. EA exerts a favorable effect on depression in patients with diabetes. This effect is reflected in the reduction in scores on the Beck Depression Inventory-II questionnaire [53]. Supplementation with EA also leads to a significant decrease in psychiatric problems, such as fatigue, anxiety, and depression, in patients with MS. Moreover, by modulating immune system activity, EA contributes to the improvement of psychological symptoms in patients with MS [55].

In contrast, treatment with urolithin A has been shown to reverse cognitive dysfunction [80]. Similarly, urolithin prevents long-term muscle weakening by specifically protecting the mitochondrial function [81]. In ocular inflammation models, urolithin A has demonstrated remarkable benefits with a favorable safety profile [82]. Urolithin B effectively alleviates depression-related behaviors, positioning it as a promising therapeutic candidate for depression through its action on neuroinflammatory pathways [83].

**Figure 2:**
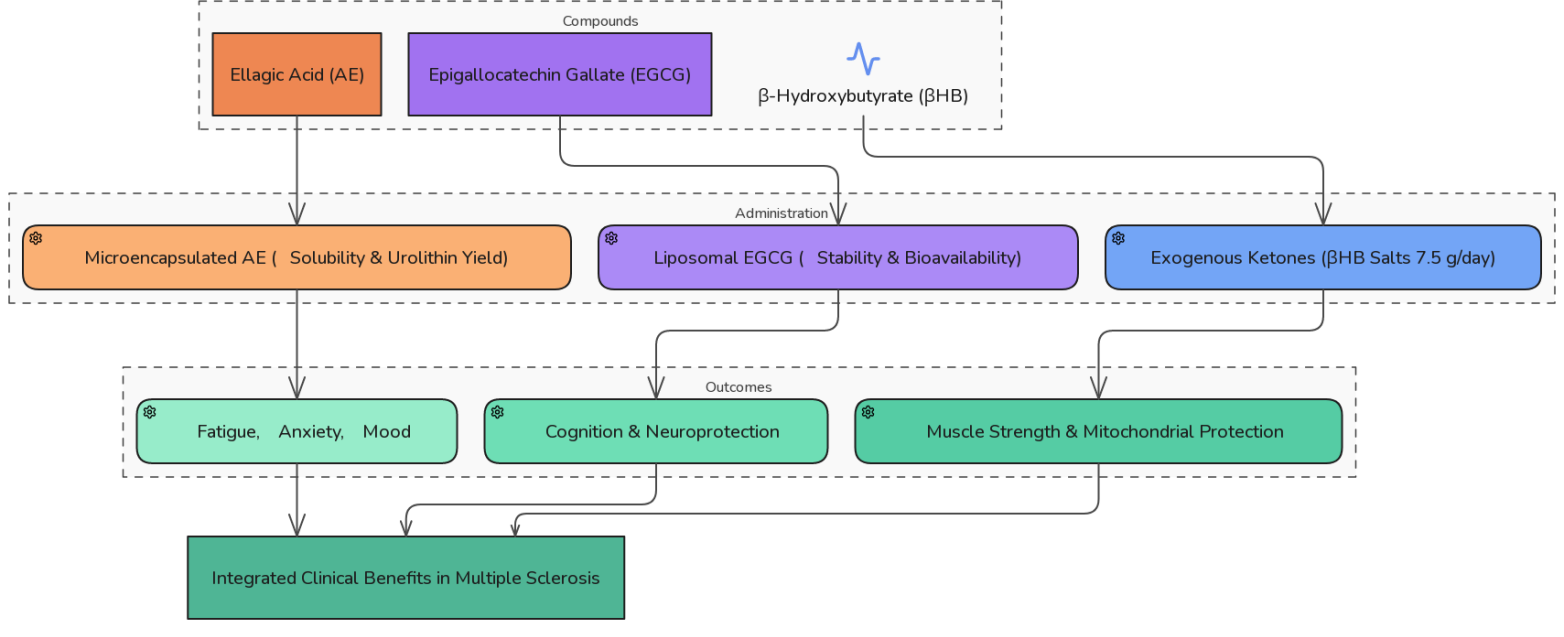
Clinical optimization of administration and efficacy of β-hydroxybutyrate, EGCG, and ellagic acid in multiple sclerosis. Schematic summary of the clinical impact, limitations, and optimized administration strategies for βHB, EGCG, and EA in multiple sclerosis. βHB improves muscle and cognitive function through metabolic regulation; EGCG enhances cognition and neuroprotection via gut-derived metabolites; and AE supports mood, mitochondrial function, and urolithin-mediated benefits. Optimized administration involves exogenous βHB salts or esters, liposomal EGCG formulations, and microencapsulated AE, each tailored to bioavailability and microbiota variability to enhance therapeutic efficacy. EA: ellagic acid; βHB: β-hydroxybutyrate; EGCG: epigallocatechin gallate.

All these clinical benefits are summarized in Figure 2.

## 6. How to Produce Ketosis and Administer EGCG and EA to Improve Efficacy

Until now, traditional methods have consisted of ketosis induction through a “ketogenic” diet high in fats and low in carbohydrates or administering MCTs in the diet containing medium-chain fatty acids (MCFAs), with oil containing 98% tricaprin being the most ketogenic [84]. However, owing to their restrictive nature and the gastrointestinal problems they cause, it can be difficult to comply with these diets or to integrate them into a daily dietary routine. This is particularly true when administering appropriate oils with corresponding proportions at the individual level. An alternative to safely increasing blood βHB concentrations is the intake of exogenous ketones [85]. Administration of exogenous ketones, such as bis-octanoyl-(R)-1,3-butanediol, allows subjects to reach the accepted threshold for nutritional ketosis (0.5 mM). This approach is generally well tolerated, produces few adverse effects, and shows good acceptance [86]. Perhaps the administration of exogenous βHB salt in doses of 7.50 g daily shows the best results in terms of safety and health metrics [87], accompanied by a diet of the same characteristics and individually adjusted to minimize differences in the degree of ketosis achieved between individuals.

Although the synergistic mechanisms of EGCG and EA polyphenols are clear at the experimental level, combined clinical studies are still limited. Interindividual variability in the bioavailability of EGCG and EA and their microbial metabolism, which is crucial, could modulate the therapeutic response. Specifically, for EA, this variability is observed in urolithin production, which has led to the stratification of volunteers into three metabotypes: metabotype A (volunteers who only produce urolithin A), metabotype B (volunteers who produce urolithin A, isoA, and urolithin B), and metabotype 0 (volunteers who do not produce urolithins) [88]. Each metabotype is associated with a specific composition of intestinal microbiota [89]. The same occurs with EGCG metabolites, whose anti-inflammatory or antidepressant activity, for example, is closely related to intestinal microbiota [74,90], as noted earlier, and especially for valerolactones, high interindividual variability has been described [91].

Microencapsulation is a plausible option for EA administration because of its low bioavailability and water solubility. Microencapsulation improves EA solubility and increases urolithin transformation in vitro [92]. Administering EGCG in liposomal form is the most effective way to improve its bioavailability, protect it from degradation throughout the gastrointestinal tract, and achieve controlled release. This approach minimizes these challenges and enhances its stability (Figure 2).

## 7. Conclusions

Mitochondrial dysfunction in complexes I, III, and IV is a central mechanism underlying neurodegeneration in multiple sclerosis. The combined administration of βHB, EGCG, and EA represents a promising multi-target strategy for addressing this energetic deficit. Each component exerts synergic effects: ketone bodies provide an alternative metabolic substrate while restoring complex I activity; EGCG primarily modulates peripheral inflammation and oxidative stress, thereby reducing secondary mitochondrial damage; and ellagic acid-derived urolithins effectively cross the blood–brain barrier to directly enhance mitochondrial biogenesis and respiratory chain function within the central nervous system.

This synergistic approach simultaneously targets energy restoration, reactive oxygen species reduction, and activation of protective epigenetic pathways. Clinical evidence supports improvements in fatigue, cognition, mood, and muscle function with these interventions. To maximize therapeutic efficacy, optimized delivery systems, including exogenous ketone salts, liposomal EGCG formulations, and microencapsulated ellagic acid, should be considered to overcome bioavailability limitations and interindividual variability in gut microbiota metabolism. Further clinical trials are warranted to validate this integrative approach in patients with MS.

## Data Availability

Primary data availability for the review are in the referenced publications.

## Abbreviations

The following abbreviations are used in this manuscript:

AMPK	AMP-Activated Protein Kinase
ATP	Adenosine Triphosphate
BBB	Blood–Brain Barrier
CNS	Central Nervous System
COX	Cytochrome c Oxidase
EA	Ellagic Acid
EAE	Experimental Autoimmune Encephalomyelitis
EGCG	Epigallocatechin-3-Gallate
ETC	Electron Transport Chain
GLUT4	Glucose Transporter Type 4
GPx	Glutathione Peroxidase
HDACs	Histone Deacetylases
HO-1	Heme Oxygenase-1
IFN-γ	Interferon-γ
IL-1β	Interleukin-1β
IL-4	Interleukin-4
IL-6	Interleukin-6
isoA	Isourolithin A
MCFAs	Medium-Chain Fatty Acids
MCTs	Medium-Chain Triglycerides
MS	Multiple Sclerosis
NAD^+^	Nicotinamide Adenine Dinucleotide (Oxidized Form)
NADH	Nicotinamide Adenine Dinucleotide (Reduced Form)
NF-κB	Nuclear Factor Kappa B
NO	Nitric Oxide
NQO1	NAD(P)H Quinone Dehydrogenase 1
NRF1	Nuclear Respiratory Factor 1
Nrf2	Nuclear Factor Erythroid-2-Related Factor 2
PGC-1α	Peroxisome Proliferator-Activated Receptor Gamma Coactivator 1-Alpha
PON1	Paraoxonase 1
ROS	Reactive Oxygen Species
SIRT	Sirtuin
SOD	Superoxide Dismutase
SOD2	Superoxide Dismutase 2
TFAM	Mitochondrial Transcription Factor A
TNF-α	Tumor Necrosis Factor-α
βHB	β-Hydroxybutyrate
